# The Role of MicroRNAs in Breast Cancer Stem Cells

**DOI:** 10.3390/ijms140714712

**Published:** 2013-07-15

**Authors:** Daniela Schwarzenbacher, Marija Balic, Martin Pichler

**Affiliations:** Division of Clinical Oncology, Department of Medicine, Medical University of Graz, Auenbruggerplatz 15, 8036 Graz, Austria; E-Mails: daniela.schwarzenbacher@medunigraz.at (D.S.); marija.balic@medunigraz.at (M.B.)

**Keywords:** microRNAs, breast cancer, tumor stem cells

## Abstract

The concept of the existence of a subset of cancer cells with stem cell-like properties, which are thought to play a significant role in tumor formation, metastasis, resistance to anticancer therapies and cancer recurrence, has gained tremendous attraction within the last decade. These cancer stem cells (CSCs) are relatively rare and have been described by different molecular markers and cellular features in different types of cancers. Ten years ago, a novel class of molecules, small non-protein-coding RNAs, was found to be involved in carcinogenesis. These small RNAs, which are called microRNAs (miRNAs), act as endogenous suppressors of gene expression that exert their effect by binding to the 3′-untranslated region (UTR) of large target messenger RNAs (mRNAs). MicroRNAs trigger either translational repression or mRNA cleavage of target mRNAs. Some studies have shown that putative breast cancer stem cells (BCSCs) exhibit a distinct miRNA expression profile compared to non-tumorigenic breast cancer cells. The deregulated miRNAs may contribute to carcinogenesis and self-renewal of BCSCs via several different pathways and can act either as oncomirs or as tumor suppressive miRNAs. It has also been demonstrated that certain miRNAs play an essential role in regulating the stem cell-like phenotype of BCSCs. Some miRNAs control clonal expansion or maintain the self-renewal and anti-apoptotic features of BCSCs. Others are targeting the specific mRNA of their target genes and thereby contribute to the formation and self-renewal process of BCSCs. Several miRNAs are involved in epithelial to mesenchymal transition, which is often implicated in the process of formation of CSCs. Other miRNAs were shown to be involved in the increased chemotherapeutic resistance of BCSCs. This review highlights the recent findings and crucial role of miRNAs in the maintenance, growth and behavior of BCSCs, thus indicating the potential for novel diagnostic, prognostic and therapeutic miRNA-based strategies.

## 1. Introduction

### 1.1. Breast Cancer and Breast Cancer Stem Cells

Breast cancer is the most frequently diagnosed cancer and the leading cause of cancer-related death among women worldwide [1]. According to the American Cancer Society, an estimated number of 232,340 new cases of breast cancer will be diagnosed in women and approximately 39,620 female breast cancer deaths are estimated in the United States for 2013. Thus, it is expected that breast cancer will account for 29% of all new cases of cancer in 2013 among women [2]. Hence, it is essential to gain a better understanding of the molecular mechanisms of breast cancer formation to ensure more efficient cancer treatments [3]. Since human breast tumors are very heterogeneous regarding time since diagnosis, histological pattern and clinical course, breast cancer can be classified into several subtypes based on distinct gene expression profiles [4]. In general, heterogeneity within and among several subtypes of cancers can arise in various ways. One common model to explain the usually observed heterogeneity of tumors is the cancer stem cell model [5]. According to the cancer stem cell hypothesis, tumors are hierarchically organized with cancer stem cells (CSCs) at the top [6] and the non-tumorigenic cell population forming the bulk of the tumor [7]. The term CSC indicates that only a subset of cancer cells in the tumor has self-renewal (asymmetric and symmetric division) capacity and the ability to produce all types of cancer cells within the tumor [6,8]. Targeting CSCs is of great interest as CSCs are considered to be more resistant to radiotherapy and chemotherapy, and are also thought to be responsible for the dissemination and growth of metastases [6].

Breast cancer stem cells (BCSCs) were originally described by Al-Hajj *et al.* in 2003. They isolated a tumorigenic subset of cancer cells from human breast tumors based on the expression of the surface markers CD44^+^, CD24^−/low^ and ESA^+^ (CD is short for cluster of differentiation, ESA is short for epithelial specific antigen).This was the first evidence for the existence of CSCs in breast cancer and they were the first to show that only the minority of breast cancer cells with a CD44^+^, CD24^−/low^ and ESA^+^ phenotype have the ability to form new tumors in NOD/SCID mice [9]. In 2007, Ginestier *et al.* indicated that high aldehyde dehydrogenase 1 (ALDH1) expression is also characteristic for BCSCs and therefore extended the BCSC phenotype on CD44^+^, CD24^−/low^, ESA^+^ and alternatively ALDH^+^[10]. Dontu and colleagues developed an *in vitro* cell culture system under non-adherent conditions for human mammary epithelial cells. Under these conditions only cells with stem cell-like properties are able to survive. These cells can proliferate and build so called mammospheres, which are multicellular formations and are thought to contain high numbers of mammary stem cells as well as progenitor cells [11]. These mammosphere cultures are commonly used in experimental studies to enrich BCSCs. However, other studies indicate that the currently used markers for BCSCs remain controversial. Lehmann *et al.* discovered that some markers used to identify putative breast tumor initiating cells do not correlate with *in vivo* tumorigenicity. Therefore it may be essential to determine other markers and/or factors that affect the increased tumorigenicity of BCSCs [12]. Another recently published study also revealed that these markers alone might not be sufficient to distinguish tumorigenic from non-tumorigenic cells. They demonstrated that tumor cells which are negative to the common CSC markers are also capable of inducing tumor growth *in vivo* [13].

### 1.2. Epithelial to Mesenchymal Transition in Cancer

Epithelial to mesenchymal transition (EMT) is an essential process during embryonic development in many species of mammals [14]. The transformation of epithelial to mesenchymal cells has also been associated with cancer progression, because the EMT program often becomes activated during cancer invasion and metastasis. This process is characterized by the loss of the epithelial marker E-cadherin, loss of cell–cell contact and cell polarity as well as an increased cell motility [15]. EMT has also been directly linked with the CSC phenotype. Induction of EMT in breast cancer cells leads to generation of cells with stem cell like properties [16].

### 1.3. Function and Biogenesis of miRNAs

It is now clear, that miRNAs together with other non-coding RNAs (long non-coding RNAs, small nucleolar RNAs and ultraconserved regions) contribute to carcinogenesis. Aberrantly expressed miRNAs are involved in initiation and progression of cancer. MiRNAs are small non-coding RNAs with a length of approximately 22 nucleotides (nt), which act as endogenous inhibitors of gene function. They modulate the expression of their target genes by either degrading their target mRNA or inhibiting their translation [17] through pairing of miRNA sequences to complementary bases on the target mRNA [18]. MiRNAs can function both as oncogenes and as tumor suppressors [19,20] and are considered as emerging potential candidates for improved cancer diagnosis, prognosis and therapy [21–24].

Biogenesis of miRNAs is a complex process. Most miRNA genes are transcribed by RNA polymerase II as long primary transcripts containing a stemloop structure. This pri-miRNA is cleaved by the RNase III endonuclease Drosha and the double-stranded RNA-binding domain (dsRBD) protein DGCR8/Pasha in the nucleus. The cleavage produces a ~70 nt hairpin precursor miRNA (pre-miRNA) with a 2-nt 3′ overhang. The 3′ overhang is recognized by Exportin-5, which transports the pre-miRNA into the cytoplasm. There, the pre-miRNA is cleaved by another RNase III endonuclease, Dicer. Dicer interacts with the dsRBD proteins TRBP/Loquacious and cleaving produces the mature ~22 nt miRNA: miRNA* duplex. The miRNA strand is usually incorporated to a RNA-induced silencing complex (RISC), a ribonuclein complex, while the miRNA* strand is typically degraded. When the miRNA is bound to RISC, the miRNA and its target mRNA can interact by base-pairing. The target mRNAs can then be cleaved and degraded or repressed in their translation [25].

In different breast cancer subtypes (basal, luminal cancers) miRNAs are differentially expressed and some miRNAs are associated with a specific ER, PR and Her2/neu status in human breast cancers [26]. MiRNAs can also function as potential targets of anticancer therapies. In breast cancer several miRNAs may possibly play a key role in cancer progression. Different studies have shown that silencing or overexpression of particular miRNAs can have an effect on the process of invasion and development of metastases in human breast cancers [27], showing a potential therapeutic application of miRNAs in breast cancer. The aim of this review is to summarize the involvement of different miRNAs in the formation and regulation of human BCSCs. Table 1 gives an overview of the roles of different miRNAs in BCSCs which are described in this review.

## 2. Particular miRNAs and Their Role in Tumor-Initiating BCSCs

### 2.1. miRNAs Down-Regulated in BCSCs

#### 2.1.1. *let-7* Family

Yu and colleagues were the first to investigate the expression of miRNAs in BCSCs in 2007. They compared the miRNA expression profile in self-renewing BCSCs and differentiated cells from breast cancer cell lines as well as in samples from primary breast tumors. They enriched BCSCs of a human breast cancer cell line (SKBR3) by passaging them in NOD/SCID mice treated with chemotherapeutical agents. The tumors contained a high percentage of CD44^+^CD24^−/low^ cells and showed high mammosphere formation ability *in vitro*. The miRNA *let-7* was found to be the most consistently down-regulated miRNA in tumor-initiating cells (SK-3rd) compared to the non-self-renewing population of cancer cells. *let-7* expression increased when the cells differentiated to non-tumorigenic cancer cells. The *let-7* family functions as a well-known tumor suppressor and targets the oncogenes rat sarcoma (RAS) and high mobility group AT-hook 2 (HMGA2). The HMGA2 gene is involved in mesenchymal cell differentiation and tumor formation. Lentiviral-mediated re-expression of *let-7* resulted in reduced mammospheres formation, proliferation and a reduced number of undifferentiated stem-like cells *in vitro. let-7* expression also inhibited the tumor formation ability in NOD/SCID mice *in vivo* and *let-7* expressing tumors had less metastatic potential. Therefore, *let-7* is apparently responsible for the regulation of multiple stem cell-like properties of BCSCs, because reduced *let-7* expression inhibits differentiation and maintains proliferation [28]. As *let-7* is a common tumor suppressor and has anti-proliferative properties, it can regulate cell differentiation and apoptotic pathways. Its down-regulation has been reported in several cancers and reconstitution of regular *let-7* expression has been shown to inhibit cancer growth [29–31]. These findings suggest *let-7* as a potential molecular marker for BCSCs with a potential as therapeutical target in anti-cancer therapy [32]. In this context, the Lin28 protein is a RNA-binding protein which regulates *let-7* family members and expression of Lin28 blocks the biogenesis of *let-7* [33]. One recently published study indicates that suppression of *let-7* through Lin28 promotes tumorigenicity in breast cancer cells [34]. Inflammatory cytokines can lead to the induction of EMT. Guo and colleagues showed that inflammatory cytokines can trigger signal transducer and activator of transcription factor 3 (Stat3) which promotes Lin28 transcription. As a consequence, this process results in repression of *let-7* expression and up-regulation of the *let-7* target HMGA2. As HMGA2 is involved in EMT, this event leads to increased levels of mesenchymal markers. These findings suggest that the inflammation-induced and Stat3 mediated Lin28-let-7-HMGA2 signaling pathway might be involved in regulation of self-renewal and differentiation in CSCs [35].

#### 2.1.2. *miR-200* Family

The *miR-200* family consists of five members of miRNAs: *miR-200a*, *miR-200b*, *miR-200c*, *miR-141* and *miR-429*. The family can be divided into genetically different subfamilies (gene clusters) according to their location at two different chromosomes: the *miR-200b*/*miR-200a*/*miR-429* gene cluster on chromosome 1 and the *miR-200c*/*miR-141* gene cluster on chromosome 12 [36]. Several recent studies have associated *miR-200* family members and their target mRNAs with establishment, maintenance and regulation of the BCSC phenotype. One of the first studies that showed an involvement of the *miR-200* family in BCSCs came from Shimono *et al.* in 2009. Comparing the miRNA expression profile between fluorescence-activated cell sorted CD44^+^CD24^−/low^ lineage BCSCs and the remaining non-tumorigenic human breast cancer cells, they found 37 miRNAs differentially expressed between non-tumorigenic cancer cells and BCSCs in eleven human breast cancer samples. They showed that three clusters of miRNAs (*miRNA-200c-141*, *miR-200b-200a-429* and *miR-183-96-182*) were consistently down-regulated in BCSCs, in normal mammary stem cells and in embryonal carcinoma cells. This finding suggests that the down-regulation of these miRNAs may be relevant for stem cell functions in cancer cells, such as self-renewal or differentiation. Downregulation of *miR-200c* in BCSCs suppressed the expression of polycomb ring finger oncogene (B lymphoma Mo-MLV insertion region 1 homolog, Bmi-1), which is a regulator of stem cell self-renewal. *miR-200c* inhibited the clonal expansion of BCSCs *in vitro*. Interestingly, *miR-200c* repressed the ability of normal mammary stem cells to generate mammary ducts and also inhibits the tumor-formation capacity of BCSCs *in vivo*. These results indicate that down-regulation of *miR-200c* might be a molecular link between CSCs and normal stem cells [37]. Consistent to that the *miR-200* family was shown to be inhibited during BCSC formation in an inducible CSC model. One of the down-regulated *miR-200* family members in BCSCs was *miR-200b* and inhibition of *miR-200b* increased the formation of BCSCs. Down-regulation of *miR-200b* resulted in increased Suz12 expression (a subunit of a polycomb repressor complex, PRC2), which led to repression of E-cadherin [38]. This inhibition of E-cadherin through miRNA is sufficient to cause EMT [39]. Overexpression of *miR-200b* or inhibition of Suz12 significantly reduced BCSC growth. In tumors of breast cancer patients, *miR-200b* and Suz12 expression were inversely correlated. Apparently the *miR-200b-*Suz12-cadherin pathway is an important pathway to induce and sustain growth of BCSCs and the invasion and migration abilities of BCSCs [38]. Another recently published study confirmed the role of the *miR-200* family in BCSCs by showing that the spontaneous conversion of immortalized human mammary epithelial cells to a stem-like phenotype with mesenchymal and less differentiated properties was accompanied by loss of *miR-200* expression. In mammospheres, *miR-200a*, *miR-200b* and *miR-200c* were described as down-regulated. Expression of *miR-200* was shown to be epigenetically regulated by histone-modifications and DNA promoter methylation. Also, in samples of pleural or ascites effusions of breast cancer patients the *miR-200* family members were consistently down-regulated in CD44^+^CD24^−/low^ putative BCSCs. Re-expression of *miR-200* in these stem-like cells led to a partial reprogramming to a non-stem like phenotype, and the cells also did undergo mesenchymal to epithelial transition (MET). These data indicate that the *miR-200* family is functionally inducing the stem-like properties [40].

#### 2.1.3. *miR-30* Family

Similar to the *let-7* family, Yu and colleagues also demonstrated the down-regulation of *miR-30*, particularly *miR-30e*, in tumor initiating BCSCs (in mammospheres SK-3rd as well as in primary BCSCs obtained from breast cancer patients). In accordance to the down-regulation of *miR-30e*, the protein levels of two direct target genes of *miR-30e*, ubiquitin-conjugating enzyme 9 (Ubc9) and integrin b3 (ITGB3), were significantly up-regulated. When *miR-30e* was constitutively expressed in BCSCs, their self-renewal capacity was impaired. This inhibition occurred through decreased Ubc9 levels and induction of apoptosis via silencing of ITGB3. Blocking of *miR-30e* in differentiated breast cancer cells on the other hand led to regeneration of their self-renewal capacity. Overexpression of *miR-30e* in NOD/SCID mice reduced tumorigenesis and lung metastases, while blocking of *miR-30e* expression enhanced tumor formation and metastases. These results indicate that reduction of *miR-30* expression is responsible for maintaining the self-renewal and anti-apoptotic features of BCSCs. *miR-30* can therefore be considered as an important miRNA for modulation of the stem-like properties of BCSCs [41]. Down-regulation of *miR-30* family members in non-adherent mammospheres compared to breast cancer cells under adherent conditions was recently confirmed in an independent study. BCSCs growth under non-attachment conditions displayed a different miRNA expression pattern compared to adherent parental cells and members of the *miR-30* family were found to be the most consistently down-regulated miRNAs in putative BCSCs. Especially *miR-30a* was found to regulate the non-attachment growth of mammospheres. Overexpression of *miR-30a* significantly reduced the mammosphere formation ability, while inhibition of *miR-30a* dramatically increased the number of mammospheres in the human breast cancer cell line MCF-7.These results confirm the relevance of this miRNA in sustaining the growth of BCSCs under non-attachment conditions. Also down-regulation of potential *miR-30a* targets after overexpressing *miR-30a* was shown. Among the potential targets, the anti-apoptotic protein AVEN was one of the most significantly down-regulated genes after overexpression of *miR-30*.This study confirms that *miR-30* family members can control expression of genes involved in apoptosis and proliferation in BCSCs [42].

#### 2.1.4. *miR-128*

The level of *miR-128* was shown to be significantly reduced in mammospheric BCSCs in two breast cancer cell lines (SK-3rd and MCF-7) and in BCSCs isolated from primary breast cancer patients. This reduction increased the protein levels of the polycomb ring finger oncogene Bmi-1 and ATP-binding cassette sub-family C member 5 (ABCC5), which are targets of *miR-128* [43]. Tumor initiating cells with stem cell-like features were shown to be more resistant to chemotherapeutical agents and radiotherapy than more differentiated tumor cells [9]. Ectopic expression of *miR-128* decreased Bmi-1 and ABCC5 levels in BCSCs and led to an enhanced pro-apoptotic and DNA-damaging effect when treated with the chemotherapeutical agent doxorubicin. This observation indicates a possible therapeutic potential of this miRNA. Furthermore, a reduction of *miR-128* in breast tumor tissues was linked with chemotherapeutic resistance and poor survival rates of breast cancer patients. Consequently the reduced levels of *miR-128* in BCSCs are likely to induce increased chemotherapeutic resistance [43]. In another study ectopic expression of *miR-128* led to a decreased number and size of mammospheres in an *in vitro* cell culture model, whereas *miR-128* depletion caused an increase of mammosphere growth. The *in vivo* tumor-initiating potential was also evaluated and it has been shown that overexpression of *miR-128-2* repressed the ability to form tumors in mice. Forced expression of *miR-128* reduced tumor growth *in vivo* and induced apoptosis [44].

#### 2.1.5. *miR-34c*

*MiR-34c* has been identified as a putative tumor suppressor and has been reported to inhibit invasion, proliferation and to promote apoptosis. Reduced expression of *miR-34c* was revealed in two human breast cancer cell lines (MCF-7 and SK-3rd) enriched for BCSCs. Down-regulation of *miR-34c* apparently occurred via hypermethylation of the promoter region of BCSCs and resulted in increased self-renewal and epithelial-mesenchymal transition of these cells. Ectopic expression of this miRNA inhibited EMT and reduced mammosphere formation and self-renewal potential. It also led to silencing of its target gene Neurogenic Locus Notch Homolog Protein 4 (Notch4) and suppressed the migration of tumor cells. This study proposed *miR-34c* as a possible target for BCSCs, as this epigenetically regulated miRNA apparently controls self-renewal and EMT in these tumor initiating cells [45].

#### 2.1.6. *miR-16*

A decreased level of *miR-16* has been shown in mammospheres derived from mammary tumors in mice compared to the whole tumor cell population. The oncogene wild-type p53-induced phosphatase 1 (Wip1) is apparently regulated by *miR-16* and protein levels of Wip1 were consequently increased in these mammospheres. Overexpression of *miR-16* in the human breast cancer cell line MCF-7 as well as inhibition of Wip1 decreased the number and size of mammospheres. When *miR-16* was overexpressed in the human breast cancer cell line MCF-7 it suppressed cell proliferation and led to an increased sensitivity to the chemotherapeutic drug doxorubicin. These findings indicate that *miR-16* is another miRNA that might be responsible for regulation of the proliferation and differentiation of mammary CSCs [46].

### 2.2. miRNAs Up-Regulated in BCSCs

#### 2.2.1. *miR-181* Family

In three human breast cancer cell lines (BT474, MDA361 and MCF7) levels of *miR-181* family members were reported to be increased in tumor initiating mammospheres compared to non-tumorigenic parental cells. Transforming growth factor-β (TGF-β) seemed to induce sphere formation by up-regulation of *miR-181* at the post-transcriptional level. A potential target of *miR-181* is the serine/threonine kinase Ataxia telangiectasia mutated (ATM) which acts as a tumor suppressor. ATM was reduced in mammospheres and after treatment with TGF-β. This study suggests that the TGF-β pathway and the *miR-181* family interacts and plays a role in regulating the BCSC phenotype [47].

#### 2.2.2. *miR-495*

Hwang-Verslues and colleagues isolated a novel highly tumorigenic subpopulation of BCSCs based on the surface markers PROCR^+^/ESA^+^ (PROCR is short for protein C receptor). In this BCSC subpopulation and also in the more commonly used CD44^+^CD24^−/low^ subpopulation, *miR-495* was highly up-regulated. As *miR-495* was found to be up-regulated in two distinct BCSC subpopulations, this mechanism might be important for maintaining stem cell-like features. This up-regulation of *miR-495* is regulated by the transcription factors E12 and E47. Overexpression of *miR-495 in vitro* promoted colony formation. *miR-495* overexpressing cells in mice led to significantly higher tumor formation *in vivo*. These results indicate that ectopic expression of *miR-495* in human breast cancer cells increases tumorigenesis *in vivo* and enhances colony formation *in vitro*. E-cadherin expression, a marker considered surrogate for EMT, was down-regulated by overexpression of *miR-495*. Decreased E-cadherin expression was responsible for promoting cell invasion. *miR-495* also targets REDD1 (short for regulated in development and DNA damage responses) which is a factor for enhanced hypoxia resistant cell proliferation. Summarizing, these findings suggest an up-regulation of *miR-495* by E12 and E47 which in turn contributes to down-regulation of E-cadherin and REDD1, finally resulting in maintaining a stem cell-like phenotype in breast cancer [48].

## 3. Conclusions

Understanding the role of miRNAs in the biology of CSCs can provide promising advances for cancer treatment and might be helpful to improve cancer diagnosis. As miRNAs are post-transcriptional regulators of gene expression, they also play important roles in carcinogenesis. Several independent studies that are reviewed here have shown a dysregulation of several different miRNAs in BCSCs. Anticancer-therapy with miRNAs could eliminate the CSC self-renewal capacity and their anti-apoptotic features which can improve the development of resistance against current cancer treatment. For this reason, future research should address the therapeutic potential of miRNAs to prevent cancer progression, relapse and formation of metastases by eliminating CSCs.

## Figures and Tables

**Table 1:** Roles of different miRNAs in BCSCs.

miRNA	Roles in BCSCs
*let-7* family	downregulated in BCSCs targets RAS and HMGA2 acts as tumor suppressor Lin28 blocks *let-7* biogenesis and promotes tumorigenic activity in breast cancer influences mammosphere formation and proliferation *in vitro* affects tumor formation ability and metastatic potential *in vivo* reduced *let-7* expression inhibits differentiation, maintains proliferation and promotes EMT
*miR-200* family	downregulated in BCSCs targets Bmi-1 and Suz12 regulation of EMT relevant for stem cell functions in cancer cells (self-renewal, clonal expansion, differentiation) *in vitro* induces stem-like properties
*miR-30* family	downregulated in BCSCs targets Ubc9, ITGB3 and AVEN influences self-renewal capacity and anti-apoptotic features important for modulation of the stem-like properties of BCSCs regulates non-attachment growth of mammospheres and mammosphere formation ability controls genes involved in apoptosis and proliferation in BCSCs
*miR-128*	downregulated in BCSCs targets Bmi-1 and ABCC5 link to chemotherapeutical resistance and survival rates of breast cancer patients influences number and size of mammospheres *in vitro* reduced tumor growth and induced apoptosis *in vivo*
*miR-34c*	downregulated in BCSCs targets Notch4 influences self-renewal and EMT acts on mammosphere formation *in vitro* is epigenetically regulated via methylation controls migration of tumor cells
*miR-16*	downregulated in BCSCs targets Wip1 influences number and size of mammospheres and cell proliferation responsible for sensitivity to chemotherapeutic drug doxorubicin
*miR-181*	upregulated in BCSCs targets ATM TGF-β induces mammosphere formation by upregulation of *miR-181*
*miR-495*	upregulated in BCSCs targets REDD1 leads to downregulation of E-cadherin promotes colony formation leads to increased tumor formation *in vivo* responsible for maintaining a stem-cell line phenotype
